# Therapeutic potential of products derived from mesenchymal stem/stromal cells in pulmonary disease

**DOI:** 10.1186/s12931-018-0921-x

**Published:** 2018-11-09

**Authors:** Arezoo Mohammadipoor, Ben Antebi, Andriy I. Batchinsky, Leopoldo C. Cancio

**Affiliations:** 1 Multi-Organ Support Technology (MOST) Task Area, US Army Institute of Surgical Research, Fort Sam Houston, TX USA; 2 Oak Ridge Institute for Science and Education, Oak Ridge, TN USA; 3 The Geneva Foundation, Tacoma, WA USA

**Keywords:** Mesenchymal stem cells, Extracellular vesicles, Conditioned media, Lung disease, Acute respiratory distress syndrome, Acute lung injury, Chronic obstructive pulmonary disease, Pulmonary fibrosis, Bronchopulmonary dysplasia

## Abstract

Multipotent mesenchymal stem/stromal cells (MSCs) possess robust self-renewal characteristics and the ability to differentiate into tissue-specific cells. Their therapeutic potential appears promising as evident from their efficacy in several animal models of pulmonary disorders as well as early-phase clinical trials of acute respiratory distress syndrome (ARDS) and chronic obstructive pulmonary disease (COPD). Such therapeutic efficacy might be attributed to MSC-derived products (the “secretome”), namely conditioned media (CM) and extracellular vesicles (EVs), which have been shown to play pivotal roles in the regenerative function of MSCs. Importantly, the EVs secreted by MSCs can transfer a variety of bioactive factors to modulate the function of recipient cells via various mechanisms, including ligand-receptor interactions, direct membrane fusion, endocytosis, or phagocytosis.

Herein, we review the current state-of-the-science of MSC-derived CM and EVs as potential therapeutic agents in lung diseases. We suggest that the MSC-derived secretome might be an appropriate therapeutic agent for treating aggressive pulmonary disorders because of biological and logistical advantages over live cell therapy. Nonetheless, further studies are warranted to elucidate the safety and efficacy of these components in combating pulmonary diseases.

## Background

The increasingly high rates of morbidity and mortality due to acute and chronic lung diseases have emerged as one of the major public health issues globally. Pulmonary diseases are mainly caused by trauma, air pollution, smoking, and various pathogens and exert a devastating effect on quality of life [1]. Acute respiratory distress syndrome (ARDS), recognized as the most severe form of acute lung injury (ALI), is a life-threatening condition featuring acute hypoxemic respiratory failure, bilateral consolidation on chest radiograph, and absence of left atrial hypertension [2, 3]. ARDS most commonly occurs due to sepsis, smoke inhalation injury, near-drowning, severe pneumonia, or pulmonary contusion [3–6]. Mechanistic studies reveal that dysregulated inflammatory/immune responses, uncontrolled activation of coagulation pathways, and increased permeability of alveolar endothelial/epithelial barrier play pivotal roles in the pathogenesis of ARDS [3]. ARDS continues to be associated with a mortality rate of 30% to 40% despite advances in modern supportive care [2, 4, 7–9].

Among many destructive chronic lung diseases, such as idiopathic pulmonary fibrosis (IPF) and pulmonary hypertension, chronic obstructive pulmonary disease (COPD) is thought to be one of the leading causes of death worldwide. Approximately 20% of COPD patients present with emphysema, which is characterized by destruction of terminal bronchioles and alveolar walls causing overdistension of air spaces due to air trapping. Despite advances in the symptomatic treatment of emphysema, a cure remains obscure [1].

Stem-cell-based therapy is an attractive approach for treating both acute and chronic lung diseases, such as ARDS and COPD, mainly because the cells can simultaneously target multiple pathological processes to protect lung function. Among the various types of stem cells, the therapeutic potential of mesenchymal stem/stromal cells (MSCs) has been extensively demonstrated in several animal models of pulmonary disorders [10–15], and is now being evaluated in phase II clinical trials for ARDS [16]. It is well established that MSCs impart their therapeutic effect via secretion of bioactive products, namely the secretome [17, 18]. Although not fully defined, the MSC secretome is comprised of an extended array of bioactive molecules that include cytokines, chemokines, growth factors, angiogenic factors, and extracellular vesicles (EVs), thus potentially contributing to the therapeutic benefits of the cells as evident from preclinical studies [19–23]. MSC secretome is readily available in the conditioned media (CM) of MSC cultures (MSC-CM) and, subsequently, MSC-derived EVs are isolated from the CM. The major advantage of MSC-derived secretome therapy over live-cell transplantation approach can be attributed to the inherent risks associated with live-cell transplants. Consequently, the MSC secretome is emerging as a viable option to replace the cells for the treatment of several lung disorders.

In this review, we describe the characteristics of MSC-CM and MSC-EVs as well as the methods of their isolation. Next, we summarize the current state-of-the-science relating to their therapeutic potential for various lung diseases. Finally, we will discuss the advantages and limitations of these products in clinical settings.

## MSC-derived products: Light at the end of the tunnel

### Mesenchymal stem/stromal cells at a glance

In 1968, Friedenstein and colleagues first discovered MSCs as a component of bone-marrow stromal tissue [24]. Subsequently, MSCs have been isolated from various tissues, such as adipose tissue, umbilical cord (UC) blood/tissue, placental tissue, and exfoliated deciduous teeth [25–29]. Since no unique surface marker(s) can exclusively identify these cells, in 2006, the Mesenchymal and Tissue Stem Cell Committee of the International Society for Cellular Therapy proposed the following criteria as minimal criteria to be satisfied for defining human MSCs. First, the cells must be plastic-adherent when maintained in standard culture conditions. Second, MSCs must express surface antigens such as CD105, CD73, and CD90 but should lack the expression of the following hematopoietic antigens: CD45, CD34, CD14 or CD11b, CD79a or CD19, and human leukocyte antigen (HLA)-DR. Third, MSCs must be capable of differentiating into osteoblasts, adipocytes, and chondroblasts in vitro [30]. That MSCs are easy to isolate and expand in culture is a major advantage from a logistical/feasibility standpoint. More importantly, MSCs have evolved as one of the mainstream cell-based therapeutic tools for regenerative medicine because of their inherent low immunogenicity and limited risk of tumorigenicity [29, 31, 32].

The initial motive for exploring their therapeutic benefits stemmed from the capability of MSCs to repair injured tissues by engrafting and replacing damaged cells through differentiation [29]. An array of other observations, however, suggests that MSCs could repair injured tissues and improve function without significant engraftment or differentiation. In fact, emerging evidence suggests that the therapeutic benefits of MSCs are derived from their secretome, and the subsequent paracrine action [29, 31–34].

### MSC Secretome

The MSC-derived secretome is released into CM in vitro [19–22, 32, 35–41]. MSCs respond to environmental changes and stress signals from injured tissues by secreting a myriad of soluble factors (Table 1); for instance, early reports by Prockop and others showed that in response to hypoxia, MSCs correlatively increase the production of several angiogenic and anti-apoptotic factors, such as interleukin-6 (IL-6), vascular endothelial growth factor (VEGF), monocyte chemotactic protein (MCP-1), also known as chemokine [C-C motif] ligand 2 (CCL2)), and stanniocalcin-1 (STC-1) [36, 37]. In line with these findings, further studies revealed that the stimulation of MSCs with different insults and inflammatory cytokines, including lipopolysaccharide (LPS), tumor necrosis factor alpha (TNF-α), IL-1β, interferon gamma (IFN-γ), and serum from ARDS patients can induce the secretion of a variety of anti-inflammatory mediators, such as TNF-stimulating gene 6 protein (TSG-6), prostaglandin E2 (PGE2), indoleamine 2,3-dioxygenase (IDO), IL-10, and IL-1 receptor antagonist (IL-1ra) [39, 42–44].

**Table 1:** Bioactive factors secreted by MSCs directly in CM or via EVs

Angiogenesis	Anti-apoptosis	Anti-fibrosis	Anti-oxidation	Chemo-attraction	Immuno- modulation	Proliferation
Ang1	FGF	Ang-1	HO-1	CCLs	HO-1	FGF
FGF	GM-CSF	FGF	IL-1β	CXCLs	IDO	HGF
HGF	HGF	HGF	STC-1	G-CSF	IL-1ra	IGF-1
IGF-1	IGF-1	KGF		LIF	IL-6	KGF
IL-6	IL-6	MMPs		M-CSF	IL-10	PDGF
MCP-1	STC-1	TIMP-1		MCP-1	LIF	VEGF
PDGF				SDF-1	PGE2	
VEGF					STC-1	
					TGF-β	
					TSG-6	

*Ang-1* – angiopoietin 1, *CCL* – chemokine ligand, *CXCL* – chemokine (C-X-C motif) ligand, *FGF* – fibroblast growth factor, *GM-CSF* – granulocyte monocyte colony stimulating factor, *HGF* – hepatocyte growth factor, *HO-1* – hemeoxygenase 1, *IDO* – indoleamine 2,3-dioxygenase, *IGF-1* – insulin like growth factor 1, *IL* – interleukin, *IL-1ra* – IL-1 receptor antagonist, *KGF* – keratinocyte growth factor, *LIF* – leukemia inhibitory factor, *LL-37* – human cathelicidin, *MMP* – metalloproteinase, *MCP-1* – monocyte chemoattractant protein 1, *PDGF* – platelet derived growth factor, *PGE2* – prostaglandin E2, *SDF-1* – stem cell-derived factor 1, *STC-1* – stanniocalcin 1, *TIMP-1* – tissue inhibitor of metalloproteinase 1, *TGF-β* – transforming growth factor beta, *TSG-6* – tumor necrosis factor-stimulated gene 6, *VEGF* – vascular endothelial growth factor

Recent efforts have focused on the paracrine effects of MSCs both in vitro and in vivo [45–52]. MSC bioactive factors have been reported to mediate several known functions of MSCs, including the modulation of immune/inflammatory responses, reduction of oxidative stress, fibrosis, and apoptosis. They also promote angiogenesis, bacterial clearance, and regeneration. In addition to their soluble factors, MSCs also secrete different types of EVs contributing to the overall therapeutic response [19–23, 40, 41].

**Fig. 1:**
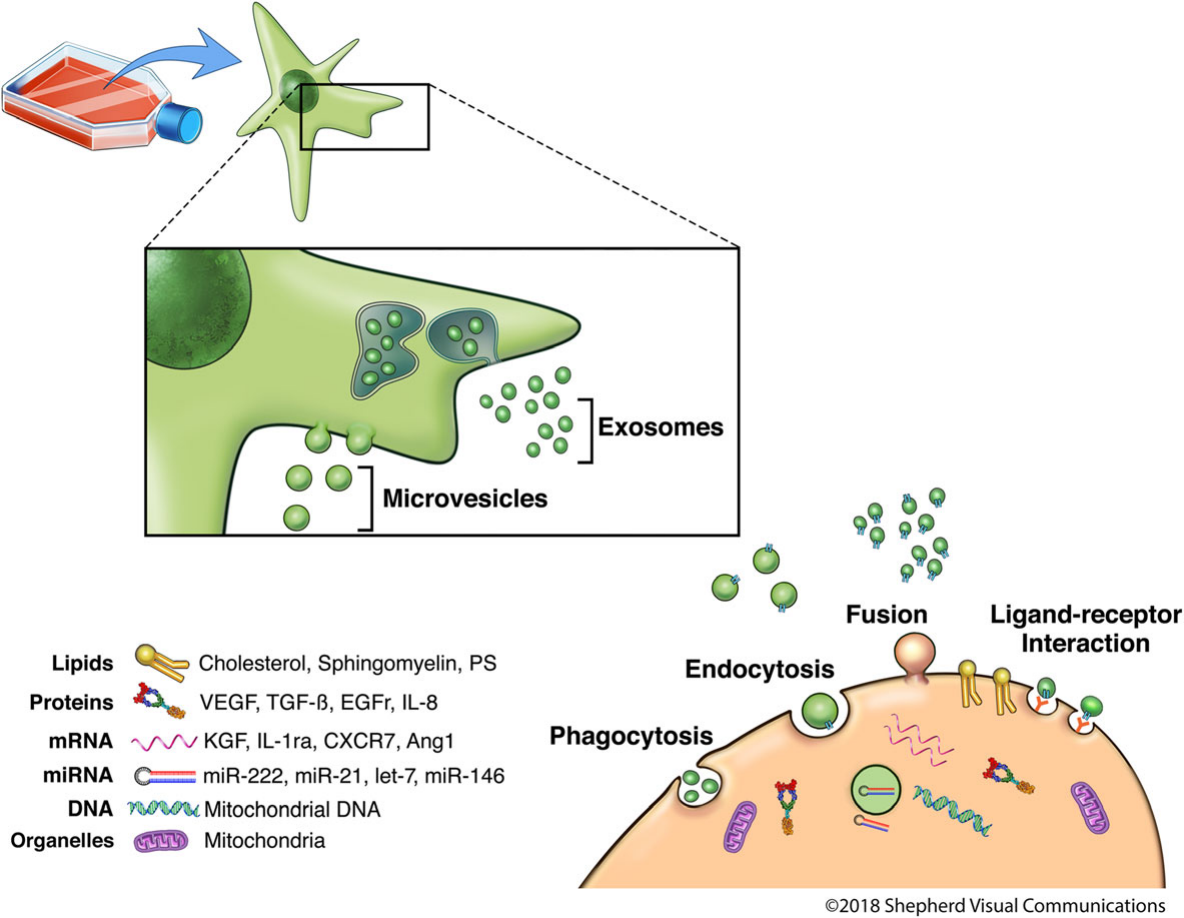
Extracellular vesicles secreted by mesenchymal stem cells transfer their cargo to the recipient cells. In culture mesenchymal stem cells secrete exosomes and microvesicles that can transfer variety of bioactive factors to the recipient cells via ligand-receptor interaction, direct membrane fusion, endocytosis, or phagocytosis. Ang1—angiopoietin 1, CXCR7 – chemokine (C-X-C motif) receptor 7, EGFr – epidermal growth factor receptor, IL-8 – interleukin 8, IL-1ra – IL-1 receptor antagonist, KGF – keratinocyte growth factor, mRNA – messenger RNA, miRNA – micro RNA, PS – phosphatidylserine, TGF-β – transforming growth factor beta, VEGF – vascular endothelial growth factor

Structurally, EVs are nano- to micro-sized particles surrounded by a phospholipid bilayer. Most eukaryotes and prokaryotes have been shown to secrete a heterogeneous population of EVs. The presence of these vesicles can be detected in physiological fluids, such as plasma, urine, cerebrospinal fluid, milk as well as in the supernatant of cell cultures in vitro [53–55]. Of note, EVs were considered as cell debris [53, 55–57] till 1996 when Raposo et al. [58] presented evidence of their biological function. They demonstrated that EVs secreted by B lymphocytes can induce antigen-specific T lymphocyte responses in vitro. The pioneering observation by these authors prompted elaborate studies to establish the role of EVs as critical mediators in cell-to-cell communication [53, 57, 59–61]. Cumulative studies in the field reveal that upon their release into the extracellular milieu, EVs can interact with recipient cells by ligand-receptor interaction or by internalization via endocytosis, phagocytosis, and direct membrane fusion (Fig. 1). Targeted delivery of EVs to specific cells/tissues is facilitated by several types of membrane molecules that are embedded in the lipid bilayers. Interestingly, several studies reported the ability of EVs to regulate a variety of biological responses in recipient cells via transfer of an array of bioactive factors that include proteins, lipids, nucleic acids (mRNA, microRNA, transfer RNA, and double-stranded DNAs), as well as cellular organelles [41, 53, 57]. At present, based on their cellular origin, secretory mechanism, size, and surface markers, EVs are classified into 3 main categories 1) exosomes; 2) microvesicles; and 3) apoptotic bodies.

*Exosomes* are formed by the inward budding of multi-vesicular bodies (MVBs), size ~ 40–100 nm, and rich in CD63, CD9, CD81, and tumor susceptibility gene 101 (Tsg 101). These vesicles are also enriched in annexins, ALG-2-interacting protein X (Alix), clathrin, heat shock proteins, and low amounts of phosphatidylserine (PS). Cytoskeleton activation is required for their release.

*Microvesicles (shedding vesicles)* are formed by budding from the plasma membrane, size ~ 80–1000 nm, and rich in integrins, selectins, and CD40 ligands. Their release is dependent on cytoskeleton activation as well as calcium influx. The lipid bilayer of microvesicles is enriched in cholesterol, sphingomyelin, ceramide, and PS.

*Apoptotic bodies* are derived from fragmentation of cells undergoing apoptosis, size ~ 1000–5000 nm, and rich in PS, nuclear fractions, and cellular organelles. While the role of cytoskeleton-associated molecules in the formation and the release of apoptotic bodies has been implicated, the mechanism responsible for relocation of fragmented DNA still remains unclear [18, 53, 62, 63].

It is now established that MSCs secrete all types of EVs [64]. The heterogeneous pool of MSC-EVs has been shown to express both EV- and MSC-specific surface markers, such as CD63, CD9, CD81, integrins, CD29, CD44, CD73, and α4-integrin [23, 65–67]. Similarly, both ubiquitous and MSC-specific proteins and nucleic acids are enveloped in these EVs (Fig. 1). Interestingly, certain proteins and nucleic acids are selectively enriched in EVs compared to their parental cells. For instance, Collino and colleagues [2010] identified that while microvesicles derived from bone marrow MSCs contain their parental cell-specific microRNAs (miR), the expression level of some was significantly higher in microvesicles compared to MSCs (miR-223, miR-451, and miR-564) [68]. It has also been shown that horizontal transfer of functional RNAs and proteins is involved in general cell functions, such as cell development, proliferation, survival, and differentiation, as well as regulation of the immune system [18], angiogenesis [69], and ATP generation [70]. Unequivocally, interest in exploring the therapeutic potential of MSC-EVs has evolved; however, at this stage, further investigation is warranted in regards to the unveiling of the composite profile of the contents of these vesicles and the elucidation of the potential therapeutic efficacy of these components.

### Preparation of MSC-CM and MSC-EVs

MSC-CM is considered the least processed cell-free product from MSCs. Based on the source of MSCs, culture condition, culture duration, and collection method, different quantities of secreted factors may be detected in the CM [50]. Generally, MSCs are cultured in their growth media as monolayers or aggregates. Once cells reach approximately 80% confluence, serum-containing growth media are replaced with fresh media [36, 47, 49, 50, 71]. In order to decrease contamination of the CM by extrinsic sources, such as fetal bovine serum, serum-free media are commonly used at this step [57]. On the other hand, chemically defined media or EV-depleted serum containing media are suggested to reduce the effect of nutrition starvation on MSCs [72]. As previously mentioned, environmental signals influence the paracrine activity of MSCs; therefore, the cells can be exposed to different stimuli, including hypoxia or pro-inflammatory cytokines to promote the secretion of certain therapeutic molecules in vitro. At the end of the incubation period (commonly 24–48 h), the supernatant is collected from the MSC cultures. The cellular component is subsequently removed by density-gradient centrifugation or filtration. At this step, the cell-free product is considered MSC-CM. It is optional to concentrate MSC-CM before in vitro and in vivo applications. Following preparation, the CM can be stored at − 80 °C or subsequently processed for EV isolation.

There are several approaches to determine the yield of the MSC-CM. One of the most common approaches is based on the number of MSCs from which the CM was derived [47, 71, 73–76]. Other parameters, including total proteins and the concentration of certain soluble bioactive mediators, have also been considered for characterization and dosage determination of MSC-CM [46, 49, 50, 77]. A variety of techniques may be used to isolate and concentrate different fractions (e.g., exosomes or microvesicles) of EVs from CM; these include ultracentrifugation, filtration and chromatography, precipitation, and immuno-affinity [78]. Each method provides different degrees of purity and enrichment; however, none of the available methods can completely separate the different types of EVs from each other or from non-EV fractions (e.g. soluble proteins, cell-free nucleic acids, and membrane fractions). Combinations of two or more methods may be used to improve the purity of certain fractions of EVs. Assessment of isolated EVs is mainly done by determination of concentration and size distributions using different methods, such as nanoparticle tracking analysis (NTA), transmission electron microscopy, and flow cytometry. In addition, the amount of total proteins in EVs is commonly assessed and used to select EV dosage for in vitro and in vivo studies. Similar to CM, the concentration of EVs or their total protein can be normalized to the number of MSCs from which they are derived. The final product can further be concentrated and stored for short periods at 4 °C. For longer storage, EVs can be placed in ≤ − 20 °C without the need of cryopreservatives.

### Therapeutic potential of MSC-CM in lung injury

So far, the ability of MSC-CM to significantly reduce the severity of lung injury, as effectively as MSCs, has been demonstrated in several in vitro and in vivo preclinical animal models (Table 2). To this end, Pati et al. showed that MSC-CM prevented endothelial cell permeability and restored the normal status of membrane adhesion molecules (β-catenin and VE-cadherin) in vitro. MSC-CM also reduced the adhesion of leukocytes to endothelial cells, thus suggesting that MSC-CM can moderate the inflammatory response of the injured endothelium by preserving vascular barrier integrity and preventing inflammatory cell binding to endothelial cells [79]. In another in vitro study, Ortiz et al. demonstrated the capability of MSC-CM to reduce the secretion of TNF-α by macrophages. Such anti-inflammatory effect of MSC-CM was suggested to be mediated by IL-1ra [10].

**Table 2:** Summary of therapeutic benefits of MSC-CM in preclinical animal models

CM Source	Route	Injury Model	Outcomes	Key Factor	Ref.
Mouse BM-MSC	IT	Mouse-ALI/LPS	↓ Neutrophils in BALF ↑ M2 in BALF	IGF-1	[74]
Human BM-MSC	IV	Rat-Pneumonia/*E. coli*	↑ Survival	LL-37	[81]
Rat BM-MSC	IT	Rat-Lung Injury/I/R	↓ Pro-inflammatory cytokines ↓ Neutrophils in BALF ↑ M2 & Treg in BALF		[82]
Mouse BMC	IN	Mouse-Asthma/OVA	↓ Airway inflammation ↓ Airway hyper-responsiveness ↓ Airway smooth muscle thickness ↑ IL-10 producing Treg & macrophages	APN	[83]
Mouse BM-MSC	IV	Neonatal Mouse-BPD/Hyperoxia	↓ Right ventricular hypertrophy ↓ Intrapulmonary arterioles muscularization ↓ Inflammatory cells & cytokines in BALF Preserve alveoli morphology ↑ Number of BASCs	Opn Csf1	[71, 84, 85]
Hyperoxia Preconditioned Rat BM-MSC	IP	Neonatal Rat- BPD/Hyperoxia	↓ PAH ↓ Right ventricular hypertrophy ↓ Pulmonary artery medial wall thickness Improved lung structure	STC-1	[87]
Rat BM-MSC	IT	Rat-Lung Fibrosis/ Bleomycin	↓ Lung fibrosis ↓ AEC apoptosis		[76]
Rat BM-MSC	IV	Rat-COPD/ cigarette smoke	↓ Lung emphysema ↓ Pulmonary artery medial wall thickness ↑ Number of small pulmonary vessels Protect lung fibroblasts		[91, 92]

*AEC* – alveolar epithelial cell, *ALI* – acute lung injury, *APN* – adiponectin, *BALF* – bronchoalveolar lavage fluid, *BASCs* – bronchoalveolar stem cells, *BM-MSC* – bone marrow-derived mesenchymal stem cells, *BPD* – bronchopulmonary dysplasia, *COPD* – chronic obstructive pulmonary disease, *Csf1* – macrophage colony stimulating factor 1 (M-CSF), E. coli – *Escherichia coli*, *IN* – intranasal, *IP* – intraperitoneally, *I/R* – ischemia reperfusion, *IT* – intratracheal, *IV* – intravenous, *LPS* – lipopolysaccharide, *M2* – macrophage type 2, *Opn* – osteopontin, *OVA* – ovalbumin, *PAH* – pulmonary artery hypertension, *STC-1* – stanniocalcin 1, *Treg* – regulatory T lymphocyte

Lee and colleagues investigated alveolar fluid transport using whole human lungs in an ex vivo lung perfusion model. The idea of such experimental studies stemmed from the notion that impaired alveolar fluid clearance is associated with higher mortality in ARDS patients. In this study, the therapeutic benefits of MSCs and MSC-CM were evaluated by the extent of their ability to restore alveolar fluid clearance in ALI. The experimental design consisted of delivery of *Escherichia coli* (*E. coli*) endotoxin intra-bronchially to perfused lungs, followed by the administration of MSCs or MSC-CM one hour following induction of injury. Both treatments improved lung endothelial barrier integrity and the rate of alveolar fluid transport. This study also revealed the essential role of keratinocyte growth factor (KGF), a MSC component, in alveolar fluid transport. MSC pretreatment with KGF small-interference RNA (siRNA) decreased the effect of MSC-CM on alveolar fluid clearance. Conversely, this effect was restored by the addition of recombinant KGF protein to the CM [80].

Further studies to elucidate the anti-inflammatory potential of MSC-CM were extended to several small animal models of ALI. In this pursuit, Ionescu et al. showed that intratracheal (IT) administration of MSCs or MSC-CM reduced the total number of infiltrated neutrophils in bronchoalveolar lavage fluid (BALF) in mice with endotoxin-induced ALI. Of note, the administration of fibroblasts or fibroblast-CM did not reproduce the same effect thus demonstrating MSC specificity. Concurrently, the flow-cytometric analysis revealed that MSC and MSC-CM administration increased the percentage of inducible nitric oxide synthase (iNOS)-Ym1^+^ alveolar macrophages in BALF, resembling the phenotype associated with alternatively activated macrophages (M2). Insulin-like growth factor 1 (IGF-1) was detected in MSC-CM, suggesting its role in polarization of alveolar macrophages to the M2 phenotype and protection of lungs from endotoxin-induced injury [74]. In another study, Devaney and colleagues reported that intravenous (IV) and IT administration of MSCs reduced the infiltration of neutrophils, IL-6, and the total amount of protein in BALF in a rat model of *E. coli*-induced pneumonia. MSCs also improved survival and bacterial clearance and preserved lung function. In contrast, MSC-CM improved animal survival without any significant mitigation of the severity of lung injury or inflammation [81]. Experimental evidence on the studies addressing the anti-inflammatory potential of MSC-CM revealed that MSC-CM pretreatment significantly reduced the amount of pro-inflammatory cytokines and the number of infiltrated neutrophils in BALF in a rat model of ischemia-reperfusion lung injury. In addition, the number of M2-like macrophages and T-regulatory lymphocytes in BALF fluid was increased in MSC-CM pretreated rats [82].

Likewise, in a mouse model of asthma, Ionescu et al. demonstrated that local delivery of CM derived from plastic-adherent bone marrow cells (BMC) is effective in reducing the number of pro-inflammatory cells in the lungs and airway hyper-responsiveness while increasing the percentage of IL-10 producing T-regulatory lymphocytes and macrophages. CM derived from lung fibroblasts did not produce the same effect as BMC-CM. Interestingly, adiponectin, an anti-inflammatory cytokine found in BMC-CM but not in fibroblast-CM, was shown to play a major role in conferring the protective effects of BMC-CM [83].

Additionally, the beneficial role of MSC-CM in protecting lung tissue from hyperoxia was studied in several small animal models of bronchopulmonary dysplasia (BPD). BPD and consequent pulmonary hypertension are serious complications in premature infants caused by mechanical ventilation and oxygen toxicity. As reported by Aslam and colleagues hyperoxia-induced BPD in neonatal mice can be prevented by IV administration of concentrated mouse MSC-CM similar to MSC treatment. This study also revealed that early administration of MSC-CM significantly decreased right ventricular hypertrophy and muscularization of intrapulmonary arterioles as well as preserved the number of alveoli with normal morphology in neonatal mice exposed to hyperoxia. In parallel, MSC-CM treatment was shown to dramatically reduce the number of inflammatory cells and cytokine levels in BALF of injured mice. Another important finding was reflected in the identification of two immunomodulatory molecules, osteopontin (Spp1 or Opn) and macrophage-colony-stimulating factor 1 (M-CSF or Csf1), exclusively present in higher extent in the MSC-CM. This suggests their plausible role in the therapeutic benefits of MSC-CM [71]. In a separate study from the same group, Hansmann observed that MSC-CM could also reverse the adverse effects of long-term exposure to hyperoxia on lung morphology and function [84]. With a view to identifying the mechanisms by which MSC-CM function, Tropea et al. observed that systemic administration of MSCs and their CM increased the number of bronchoalveolar stem cells (BASCs) in terminal bronchioles of lungs of neonatal mice 10 days post administration. Similarly, MSC-CM enhanced BASC colony growth in vitro; however, supplementation of BASC cultures with growth factors known to be present in MSC-CM (VEGF, HGF, KGF, or basic FGF) did not show a similar effect, thus ruling out the association of these MSC components in enhanced BASC colony growth [85]. It is noteworthy that the effect of daily administration of human MSC-CM, intraperitoneally, was observed to be comparable with a single administration of MSCs in protecting neonatal rats from BPD and pulmonary hypertension [86]. Since culture conditions can influence the secretion pattern of MSCs, Waszak and colleagues examined the effect of hyperoxia on the therapeutic potential of preconditioned MSC-CM in a rat model of BPD. Preconditioning MSCs under 95% O_2_ improved therapeutic benefits of the CM compared with control MSC-CM or lung fibroblast-CM. Correlatively, the preconditioned MSC-CM was also shown to contain a higher amount of STC-1 [87], which is a pleiotropic protein with antioxidant, anti-apoptotic, and anti-inflammatory potential [37, 88–90].

Shen and colleagues examined the anti-apoptotic and anti-fibrotic benefits of MSC-CM in a chronic model of bleomycin-induced lung injury. Histological analysis of MSC-CM-treated rats revealed a significant reduction in lung fibrosis and apoptosis 28 days post-injury. Interestingly, the MSC-CM was able to promote proliferation and prevent apoptosis of human non-small cell lung cancer epithelial cells in vitro [76].

The therapeutic benefits of MSC-CM were also evaluated in a small animal model of cigarette-smoke-induced COPD. In addition to lung emphysema, cigarette smoke can cause apoptosis in lung fibroblasts. One of the roles of lung fibroblasts is to maintain the integrity of the alveolar structure; thus, their normal function is essential for the regeneration of the injured lung. Huh et al. showed that MSC and MSC-CM treatment significantly improved the histology of the emphysematous lungs and increased the number of small pulmonary vessels [91]. In a separate study, Kim et al. reported that MSC-CM reduced lung fibroblast apoptosis and increased their proliferation in vitro and in vivo. MSC-CM also restored the expression of extracellular matrix proteins and collagen gel contraction mediated at least in part by the PI3K/Akt pathway. Interestingly, the presence of both anti- and pro-fibrotic mediators was detected in MSC-CM [92].

Overall, these reports strongly suggest that administration of CM derived from MSCs can significantly attenuate cell death and inflammatory responses while improving tissue healing and endogenous regeneration in various lung injury models. However, the key mediators and the optimal conditions to produce them are still largely unknown.

### Therapeutic potential of MSC-EVs in lung injury

Among the potential key mediators of MSCs, MSC-EVs are considered to bear therapeutic potential in lung injury. In order to establish a safe and effective alternative to live-cell transplantation, the therapeutic potential of different EV fractions was examined in a variety of preclinical animal models of lung injury as summarized in Table 3.

**Table 3:** Summary of therapeutic benefits of MSC-EVs in preclinical animal models

EV Source	Route	Injury Model	Outcomes	Key Factor	Ref.
Human BM-MSC	IT, IV	Mouse-ALI/LPS	↓ Lung edema ↓ WBCs & neutrophils in BALF ↓ Total protein & MIP-2 in BALF ↑ KGF in BALF	KGF mRNA	[22]
Normoxia & Anoxia pretreated Human BM-MSC	IV	Mouse-ALI/LPS	↓ WBCs & neutrophils in BALF ↓ Total protein & MIP-2 in BALF		[94]
Untreated & Poly (I:C)–pretreated Human BM-MSC	IT, IV	Mouse-Pneumonia/*E. coli*	↓ Lung injury ↓ WBCs & neutrophils in BALF ↓ Total protein & MIP-2 in BALF ↓ E. coli count in BALF, lung, & blood ↑ Survival ↑ KGF in BALF	KGF mRNA & CD44	[95]
Human BM-MSC	EV-treated AM/IN	Mouse-ALI/LPS	↓ Neutrophils in BALF ↓ Total protein & TNF-α in BALF	Mitochondria transfer	[96]
Human BM-MSC	IV	Mouse-Shock/Hemorrhage	↓ Vascular permeability ↓ RhoA GTPase activity in lung		[66]
Human WJ-MSC	IT	Mouse-Lung Injury/I/R	↓ Lung edema ↓ Airway resistance ↓ Pulmonary artery pressure ↓ Neutrophil in lung ↓ Inflammatroy cytokines in BALF ↑ KGF, PGE2, &IL-10 in BALF		[115]
Swine BM-MSC	IT	Swine-Influenza/SwIV	↓ Lung lesions ↓ WBCs in lung ↓ Inflammatory cytokines in lung ↓ Virus titer in nasal swap & lung	RNA	[101]
Mouse & Human BM-MSC	IV	Mouse-Asthma/AHE	↓ Airway hyper-responsiveness ↓ WBCs in BALF & lung ↓ Th2/Th17 related cytokines in BALF		[75]
Mouse BM-MSC	IV	Mouse-PAH/Hypoxia	↓ Right ventricular systolic pressure ↓ Vascular remodeling ↓ Macrophages & cytokines in lung ↓ STAT-3 in lung		[20]
Rat BM-MSC	IV	Rat-PAH/Monocrotaline	↓ Pulmonary artery pressure ↓ Pulmonary vascular remodeling ↓ Right ventricle pressure ↓ Right ventricular hypertrophy		[98]
Mouse & Human BM-MSC	IV	Mouse-PAH/ Monocrotaline	↓ Pulmonary vascular remodeling ↓ Right ventricle hypertrophy	miRNAs	[99]
Human WJ- & BM-MSC	IV	Neonatal Mouse-BPD/Hyperoxia	↑ Alveolarization ↑ Lung funcion ↓ Lung fibrosis ↓ PAH ↓ Pulmonary vasular remodeling ↓ Pro-inflammatry genes in macrophages		[67]
Human UC-MSC	IT	Neonatal Rat-BPD/Hyperoxia	↑ Alveolarization & angiogenesis ↓ Alveolar epithelial cell death ↓ Macrophages & cytokines in lung	VEGF	[100]
Human BM-MSC	IV	Mouse-Lung Fibrosis/Silica	↓ Size of calcified nodules in lung ↓ Hydroxyproline in lung ↓ Inflammatory cells in BALF ↓ Cytokines in BALF	miRNAs & mitochondria transfer	[17]
Human BM-MSC	IV	Mouse-Lung Fibrosis/Silica	↓ Lung collagen ↓ WBCs in BALF		[97]
Human ASCs	IT	Mouse-COPD/ elastase	↓ Lung emphysema ↑ FGF2 in lung		[116]

*AHE* – *Aspergillus* hyphal extract, *ALI* – acute lung injury, *AM* – Alveolar macrophages, *ASCs* – adipose-derived mesenchymal stem cells, *BALF* – bronchoalveolar lavage fluid, *BM-MSC* – bone marrow-derived mesenchymal stem cells, *FGF2* – fibroblast growth factor 2, *IL-10* – interleukin 10, *IN* – intranasal, *I/R* – ischemia reperfusion, *IT* – intratracheal, *IV* – intravenous, *KGF* – keratinocyte growth factor, *LPS* – lipopolysaccharide, *MIP-2* – macrophage inflammatory protein 2, *miRNAs* – microRNAs, *OVA* – ovalbumin, *PAH* – pulmonary artery hypertension, *PGE2* – prostaglandin E2, *STAT-3* – signal transducer and activator of transcription 3, *SwIV* – swine influanza virus H1N1, *Th* – T helper lymphocyte, *UC-MSC* – umbilical cord blood-MSC, *VEGF* – vascular endothelial growth factor, *WBCs* – white blood cells, *WJ-MSC* – umbilical cord Wharton’s jelly-MSC

Gennai and colleagues used an ex vivo lung perfusion model to demonstrate the effect of MSC-EVs on alveolar fluid clearance rate in human lungs rejected for transplantation. MSC-EVs improved alveolar fluid clearance rate in a dose-dependent manner. Lung weight gain was also reduced by MSC-EV therapy compared to perfusion alone. Conversely, EVs derived from normal human lung fibroblasts had no beneficial effects. Further, MSC-EVs restored tracheal pressure, improved lung compliance, and reduced pulmonary artery pressure over six hours. The amount of lactate was also significantly lower in MSC-EV treated lungs compared to the control lungs. Since the anti-CD44 antibody attenuated the uptake of EVs by alveolar epithelial cells, the study suggested a key role of the CD44 receptor in internalizing EVs into recipient cells [93].

The studies on exploring therapeutic benefits of MSC-EVs have been examined in several ALI models. In this regard, an investigation by Zhu and colleagues recently showed that IT administration of MSC-EVs was associated with a reduction in pulmonary edema in *E. coli* endotoxin-induced ALI in mice. Additionally, MSC-EVs attenuated the influx of inflammatory cells and the presence of macrophage inflammatory protein 2 (MIP-2) in the alveoli. Both IT and IV administration of MSC-EVs were shown to dramatically increase the amount of KGF in BALF compared to injured untreated mice, maybe via transfer of KGF mRNA [22]. In another study, Li et al. preconditioned MSCs under anoxia for 60 min prior EV collection. Subsequently, EVs from both normal culture and preconditioned MSCs were observed to significantly reduce the total number of white blood cells, neutrophils, total proteins, and MIP-2 in BALF of endotoxin-injured mice; however, no significant differences were observed between the preconditioned MSC-EVs and control MSC-EVs [94].

Another report by Monsel and colleagues demonstrated that systemic administration of MSC-EVs improved survival and reduced lung inflammation and injury while promoting bacterial clearance in a murine model of *E. coli*-induced pneumonia. EVs harvested from MSCs preconditioned with a toll-like receptor (TLR)-3 agonist were shown to further increase KGF secretion and bacterial clearance. When examined in vitro, the MSC-EVs increased the phagocytic function of human macrophages and the ATP levels in alveolar epithelial type 2 cells. Preconditioned MSC-EVs were more effective in enhancing the anti-inflammatory and phagocytic activity of cultured macrophages, possibly by transferring cyclooxygenase-2 mRNA to activated monocytes resulting in an increase in production of PGE2 [95].

The direct effects of MSC-EVs on activated macrophages has also been studied by preconditioning alveolar macrophages (AMs) with MSC-EVs and administering them to mice with LPS-induced lung injury. Administration of pretreated AMs, but not control AMs, significantly reduced total proteins, TNF-α, and the total number of neutrophils in BALF. Mitochondrial transfer via MSC-EVs was shown to play an important role in carrying the anti-inflammatory and pro-phagocytic effects of MSCs on macrophages [96]. Phinney and colleagues demonstrated that under oxidative stress, MSCs donated mitochondria to macrophages via EVs. In addition, they revealed that IV administration of MSCs or their EVs in silica-exposed mice significantly reduced the infiltration of white blood cells and secretion of inflammatory mediators in BALF. MSCs and their EVs also reduced the size of the calcified nodules as well as the expression of pro-inflammatory [TNF, CCL2, and chemokine (C-X-C motif) ligand (CXCL) 1] and pro-fibrotic (TGF-β and IL-10) genes in the lung, 2 to 4 weeks post-injury. Interestingly, MSC-EVs, but not MSCs alone, reduced the accumulation of hydroxyproline in lung tissue [17]. Systemic administration of MSCs or EVs following silica exposure was shown to decrease collagen deposition in lung parenchyma and recruitment of neutrophils and lymphocytes into airways two weeks post-treatment. However, MSC-EV administration did not significantly reduce lung wet-to-dry ratio, as opposed to MSCs [97].

The therapeutic benefits of MSC-CM and MSC-EVs were compared in a mouse model of acute asthma. Systemic administration of both MSC-CM and EVs, but not lung fibroblast products, significantly abrogated the increase in airway hyper-responsiveness and lung inflammation. The amounts of T helper lymphocyte (Th)2- and Th17-associated cytokines were reduced in BALF while IL-10 levels were concomitantly increased. Blocking the release of secreted mediators in MSCs in vitro suppressed many protective effects of the cells [75].

In a murine model of hemorrhagic shock, MSCs and their EVs were shown to attenuate vascular permeability in injured lungs. Both treatments were able to suppress hemorrhagic-shock-induced RhoA GTPase activity in lungs. MSC-CM and recombinant angiopoietin 1 (Ang1), but not EVs, significantly protected endothelial VE-cadherin junctions and reduced permeability in vitro. In vivo analysis of protein phosphorylation patterns also revealed some differences in signaling pathways activated by MSC-EVs compared to MSCs. Overall, these observations suggest that while MSC-EVs preserved pulmonary vasculature integrity similar to MSCs, their mechanism of action was not necessarily equivalent [66].

The effect of MSC-EVs on pulmonary vasculature was also demonstrated by Lee and colleagues. Studies by this group indicated that the administration of MSC-EVs, but not fibroblast-EVs, can protect against the elevation of right ventricular systolic pressure and pulmonary vascular remodeling in a murine model of hypoxia-induced pulmonary artery hypertension. The MSC-EVs mediated an anti-inflammatory effect by reducing the influx of lung macrophages and early inflammatory mediators. MSC-EV treatment also abrogated the hypoxia-induced phosphorylation of signal transducer and activator of transcription 3 (STAT-3), which plays a critical role in the response of pulmonary vasculature to hypoxia [20]. In a rat model of monocrotaline-induced pulmonary hypertension, IV administration of rat MSC-EVs decreased pulmonary artery pressure and remodeling as well as right ventricle pressure and hypertrophy. MSC-EVs were as effective as their parent cells in preventing pulmonary hypertension [98]. Similarly, IV administration of either mouse or human MSC-EVs was shown to reduce right ventricular hypertrophy and pulmonary vascular remodeling in monocrotaline-treated mice. Administration of the exosome fraction of MSC-EVs exerted higher therapeutic response compared to the microvesicle fraction. MicroRNA analysis revealed a significant increase in the levels of several anti-inflammatory and anti-proliferative microRNAs, including miRs-34a,-122,-124, and − 127 in MSC-exosomes compared to exosomes derived from plasma of healthy mice [99].

Similar to MSC-CM, MSC-EVs were shown to significantly reduce lung fibrosis, restore lung architecture, and improve alveolarization and lung function of neonatal mice exposed to 75% oxygen for 7 days. MSC-EV administration also reduced pulmonary arterial remodeling and hypertension associated with BPD. Additionally, MSC-EVs downregulated expression of genes associated with pro-inflammatory macrophages [67]. In a study conducted by Ahn and colleagues, EVs derived from UC-MSCs were as effective as their parental cells in preventing hypoxia-induced BPD in neonatal rats. Also, MSCs and their EVs reduced the inflammatory responses in the lungs in contrary to fibroblast-derived EVs that did not exhibit similar protective effects. Finally, the protective effect rendered by MSCs and EVs was partially abrogated by the knockdown of the VEGF gene in MSCs prior to EV isolation [100].

Recently, MSC-EVs were examined in a swine model of influenza virus-induced ALI. Local administration of MSC-EVs was able to significantly reduce the infiltration of inflammatory cells to the lungs, the thickening of alveolar walls, and the number of collapsed alveoli in infected swine. MSC-EVs were also able to reduce replication and shedding of influenza virus both in vivo and in vitro. It was suggested that the transfer of RNA to the infected cells might be an important factor in the observed anti-influenza activity by MSC-EVs [101].

Overall, cumulative studies described above suggest that in order to increase cell survival, preserve physiological function, and reduce immune/inflammatory responses, MSC-EVs can transfer bioactive mediators to injured cells and regulate their pathophysiologic responses. In most experiments, EVs appeared to be as potent as MSCs, possibly with lower health-associated risks.

### MSC-EVs: From bench to bedside

In 2014, allogeneic MSC-EVs were administered to a patient with a steroid refractory graft-versus-host disease (GvHD). Not only the diarrhea volume of the patient was reduced after MSC-EV therapy, but also cutaneous and mucosal symptoms were improved within two weeks and the patient was in stable condition four months after EV treatments. Consequently, the dose of steroids given to the patient was reduced. Interestingly, the number of patient’s peripheral blood mononuclear cells producing TNF-α, IL-1β, or IFN-γ was reduced more than 50% after the last EV administration [102].

In a separate study, forty patients with chronic kidney disease (CKD) were randomly divided into two groups to receive MSC-EVs or placebo treatment. Two doses of MSC-EVs were administered a week apart. The first dose was administered IV and the second intra-arterially. Of note, MSC-EV treatment, but not the placebo, was effective in improving estimated glomerular filtration rate and urinary albumin-to-creatinine ratio as well as reducing serum creatinine and blood urea. At twelve weeks after EV treatment, plasma levels of TNF-α were significantly reduced, whereas levels of IL-10 and TGF-β were increased [103]. In both studies, the MSC-EV administration was well-tolerated by patients without any side effects.

A phase II/III clinical trial is currently registered on clinicaltrials.gov evaluating the benefits of UC-MSC-EVs in type I diabetic patients [104]. In addition, recently, an Investigational New Drug clearance for allogeneic MSC-EVs was approved by the Food and Drug Administration for the treatment of severe second-degree burn patients. The company that received the approval is planning to conduct a multicenter phase I/IIa clinical trial in late 2018 [105].

## Conclusions

Overall, current data suggest that MSC-derived products can effectively mimic the therapeutic effects of MSCs in preclinical models of lung injury. The use of CM or EVs may circumvent many of the safety concerns associated with the use of live cells, such as thrombogenicity. Also, unlike cells, CM and EVs are not able to proliferate or reprogram after administration, which decreases the risk of tumorigenicity [106–108]. From a therapeutic standpoint, MSC-EVs and other bioactive mediators are smaller than their parent cells, and thus might be distributed more efficiently into the lungs and other organs.

Another therapeutic advantage of EVs arises from their ability to protect their cargo from unfavorable environmental conditions, such as dramatic changes in pH or release of digestive (lytic) enzymes into the bloodstream and damaged tissue. Based on this concept, MSC-EVs are considered efficient vehicles for transporting delicate bioactive mediators to the distal airways. It is also thought that MSC-EVs can be used to deliver specific drugs to target organs. Such drugs can be delivered to EVs via MSCs that overexpress those factors, or can be loaded directly to the EVs after they have been released by the cells. Examples of different types of therapeutic cargo include siRNAs for silencing target genes and chemotherapeutic drugs, such as doxorubicin [109, 110].

In addition to their biological advantages, feasibility/logistical advantages set cell-free products apart. For instance, large-scale manufacturing of cell-free products is possible by repeated harvesting of CM from cultured MSCs. MSC-CM and EVs can be prepared and stored in small volumes, which could simplify dosing studies. Another great advantage is that CM and EVs can also be stored for several months at − 20 °C to − 80 °C without the need for cryopreservatives, unlike their parent cells [111]. Notably, cryopreservatives, such as dimethyl sulfoxide (DMSO), can be cytotoxic and have been reported to lyse EVs [112]. The potential capability to freeze-dry and lyophilize all or pertinent fractions of CM and EVs should be helpful in overcoming the logistical difficulties of storage and transport of these products in austere conditions.

There appear to be therapeutic advantages in using MSC-CM and EVs over live whole cells; yet, to develop safe and effective cell-free therapies, a significant knowledge gap needs to be addressed, including optimization of bioactive components, dosing regimens, and production methods. A growing number of studies have identified some of the key mediators in MSC-CM and EVs; however, there is a variability in the content of the secretome due to several factors. These include donor’s condition (age, sex, and health status), the type of MSCs (e.g. bone marrow, adipose, or UC), the type of culture (flask or bioreactor), the type of media and supplements (e.g., fetal bovine serum, xeno-free, or chemically-defined media), and the microenvironment (e.g. oxygen tension and presence or absence of stress signals) [16, 104, 111]. Also, post-processing of CM and EVs (e.g. CM concentration and EV enrichment) as well as handling and storage can influence their quality [111]. Furthermore, various factors (e.g. methods of EV enrichment and storage) could influence the quantity of EVs present in the final product [104], making it difficult to estimate EV yield per MSC. In light of these limitations, it is essential to develop a series of potency assays for cell-free products in order to control their quality and quantity, and to predict their efficacy in vivo.

Based on currently available techniques, large numbers of MSCs are still required to produce an adequate amount of CM or EVs for clinical use, similar to the number of MSCs used for clinical trials (e.g. 10^6^–10^7^ cells/kg). In order to produce sufficient pools of MSCs, the cells need to be expanded in vitro for several population doublings. Although MSCs have been shown to be safe for up to 40 population doublings [16], it is important to frequently inspect them for cell senescence and chromosome instability, and eliminate defective cells. To overcome the low yield of MSCs and their products from primary cultures, genetic modification, such as immortalization, can be considered [113]. In addition, the technology to obtain CM and EVs from MSCs derived from induced pluripotent stem cells (iPSC-MSCs) might be beneficial [111, 114]. iPSC-MSCs have robust proliferation and differentiation capacities and can be generated either from allogeneic sources or patients [114]. These alternative approaches could also facilitate establishing universal donors to minimize donor-to-donor variability and produce large-scales of CM and EVs with consistent quality. The use of autologous MSCs and their products may be considered when allogeneic products are not available, especially in remote and austere conditions. However, since the effect of different disorders on endogenous stem cells and their products is not completely understood [16, 111], the application of autologous-based products needs to be carefully assessed.

To summarize, MSC-CM and EVs emerge as promising therapeutic tools for the treatment of acute and chronic lung diseases. However, there is a compelling need to gather additional knowledge. Furthermore, large-scale production of Good Manufacturing Practice (GMP)-graded MSC-CM and EVs needs to be developed so that cell-free therapy can be safely translated from the bench to the bedside.

## Abbreviations

AEC: Alveolar Epithelial Cell
AHE: *Aspergillus* Hyphal Extract
ALI: Acute Lung Injury
Alix: Apoptosis-Linked Gene (ALG)-2-Interacting Protein X
AM: Alveolar Macrophages
Ang1: Angiopoietin 1
APN: Adiponectin
ARDS: Acute Respiratory Distress Syndrome
ASC: Adipose-derived Mesenchymal Stem/stromal Cell
BALF: Bronchoalveolar Lavage Fluid
BASCs: Bronchoalveolar Stem Cells
BMC: Bone Marrow Cells
BPD: Bronchopulmonary Dysplasia
CCL: Chemokine (C-C motif) ligand
CKD: Chronic kidney disease
CM: Conditioned Media
COPD: Chronic Obstructive Pulmonary Disease
CXCL: Chemokine (C-X-C motif) ligand
CXCR: Chemokine (C-X-C motif) Receptor
DMSO: Dimethyl sulfoxide
E.coli: *Escherichia coli*
EGFr: Epidermal Growth Factor Receptor
EVs: Extracellular Vesicles
FGF: Fibroblast Growth Factor
G-CSF: Granulocyte Colony Stimulating Factor
GM-CSF: Granulocyte Monocyte Colony Stimulating Factor
GMP: Good Manufacturing Practice
GvHD: Graft-versus-Host Disease
HGF: Hepatocyte Growth Factor
HLA-DR: Human Leukocyte Antigen
HO-1: Hemeoxygenase 1
I/R: Ischemia Reperfusion
IDO: Indoleamine 2,3-Dioxygenase
IFN-γ: Interferon Gamma
IGF-1: Insulin-like Growth Factor 1
IL: Interleukin
IL-1ra: IL-1 Receptor Antagonist
iPSC-MSC: Induced Pluripotent Stem Cell-derived Mesenchymal Stem/stromal Cell
IN: Intranasal
iNOS: Inducible Nitric Oxide Synthase
IPF: Idiopathic Pulmonary Fibrosis
IT: Intratracheal
IV: Intravenous
KGF: keratinocyte Growth Factor
LIF: Leukemia Inhibitory Factor
LPS: Lipopolysaccharide
M2: Alternatively Activated Macrophages
MCP-1: Monocyte Chemotactic Protein 1
M-CSF (Csf1): Macrophage-Colony Stimulating Factor 1
MIP-2: Macrophage Inflammatory Protein 2
miRNA (miR): MicroRNA
MMP: Metalloproteinase
mRNA: Messenger RNA
MSCs: mesenchymal stem/stromal cells
MVBs: Multi-Vesicular Bodies
NTA: Nanoparticle Tracking Analysis.
Opn: (Spp1) Osteopontin.
OVA: Ovalbumin.
PAH: Pulmonary Artery Hypertension.
PDGF: Platelets Derived Growth Factor.
PGE2: Prostaglandin E2.
PS: Phosphatidylserine.
SDF-1: Stem Cell-Derived Factor 1.
siRNA: Small Interference RNA.
STAT-3: Signal transducer and activator of transcription 3.
STC-1: Stanniocalcin-1.
SwIV: Swine Influenza Virus H1N1.
TGF-β: Transforming Growth Factor beta.
Th: T Helper Lymphocyte.
TIMP-1: Tissue Inhibitor of Metalloproteinase 1.
TLR: Toll-like Receptor.
TNF-α: Tumor Necrosis Factor Alpha.
Treg: Regulatory T Lymphocyte.
Tsg 101: Tumor Susceptibility Gene 101.
TSG-6: TNF Stimulating Gene 6 Protein.
UC-MSC: Umbilical Cord Blood Mesenchymal Stem/stromal Cell.
VEGF: Vascular Endothelial Growth Factor.
WBC: White Blood Cell.
WJ-MSC: Umbilical Cord Warton’s Jelly Mesenchymal Stem/stromal Cell.
